# ﻿Notes on the green lacewing subgenus Chrysopidia (*s. str.*) Navás, 1910 (Neuroptera, Chrysopidae), with description of a new species from China

**DOI:** 10.3897/zookeys.1106.83232

**Published:** 2022-06-16

**Authors:** Yunlong Ma

**Affiliations:** 1 Engineering Research Center for Forest and Grassland Disaster Prevention and Reduction, Mianyang Normal College, Mianxing West Road, 621000, Mianyang, China Engineering Research Center for Forest and Grassland Disaster Prevention and Reduction, Mianyang Normal College Mianyang China

**Keywords:** Key, new species, taxonomic study

## Abstract

A taxonomic study of the green lacewing subgenus Chrysopidia (*s. str.*) from China is presented. Based on the examination of type specimens of the genus reported by previous Chinese scholars and the line drawings of *Chrysopidiamanipurensis* provided by Ghosh (1990), I proposed five new combinations: *Apertochrysaplatypa* (Yang & Yang, 1990), **comb. nov.**, *Apertochrysayangi* (Yang, 1997), **comb. nov.**, *Apertochrysashennongana* (Yang & Wang, 1990), **comb. nov.**, *Apertochrysazhaoi* (Yang & Wang, 1990), **comb. nov.**, and *Apertochrysamanipurensis* (Ghosh, 1990), **comb. nov.** As for *Chrysopidiasinica* Yang & Wang, 1990, I still treat it as a valid species until supplementary specimens are available because the gonarcal complex of the type specimen is missing. A new species Chrysopidia (Chrysopidia) tjederi**sp. nov.** is also described based on new materials. A key to all known species of this subgenus is also provided.

The paper has been retracted by the publisher due to evidences of misconduct.

The removed version is archived for the purpose of resolving nomenclatural questions and is available from the publisher upon request.

